# Supplementary figures and images for: Distinct patterns of brain Fos expression in Carioca High- and Low-conditioned Freezing Rats
Source: PLoS One. 2020 Jul 23;15(7):e0236039. doi: 10.1371/journal.pone.0236039 (PMC7377485; doi:10.1371/journal.pone.0236039)

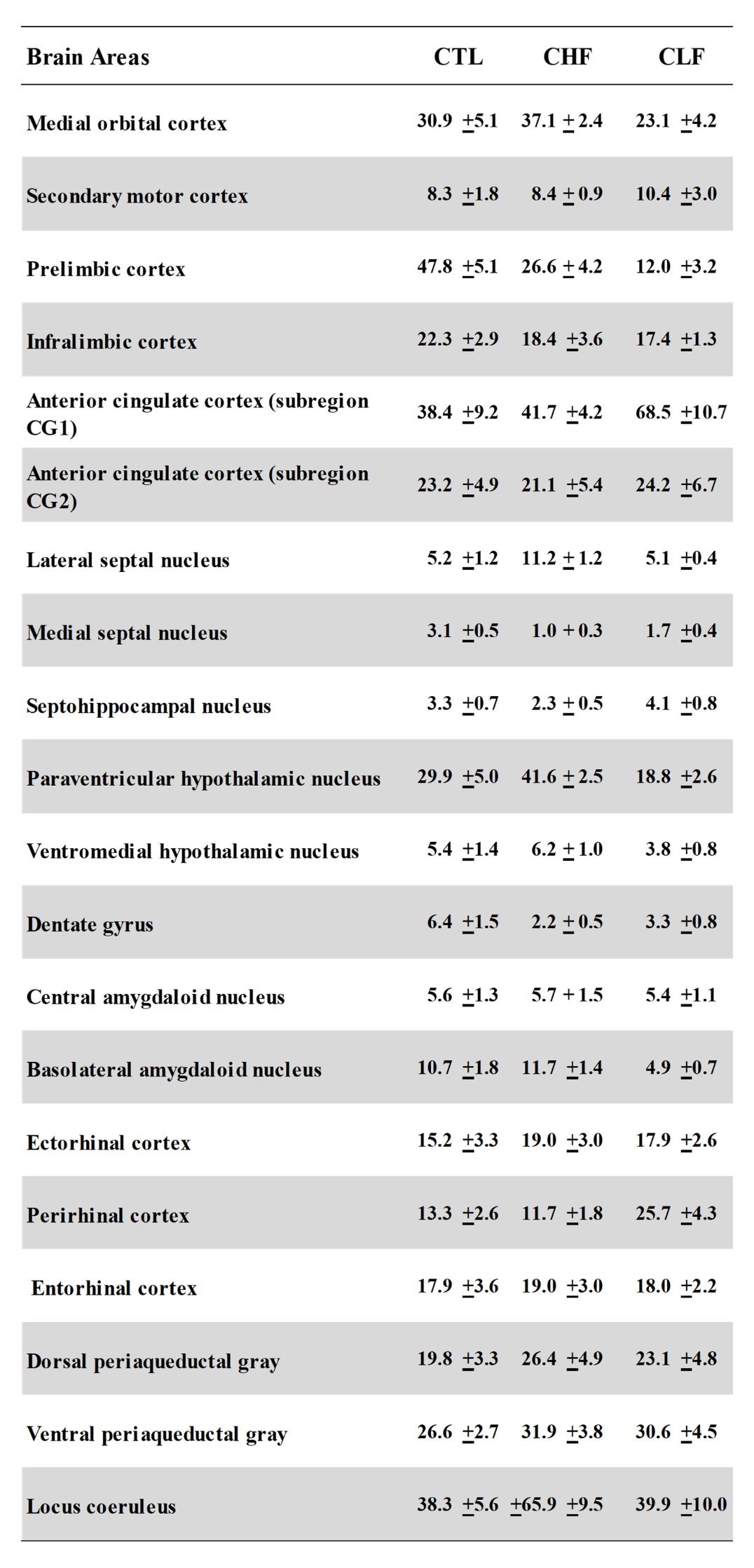

Supplement: S1 Table — (DOCX) [file pone.0236039.s001.docx]
