# Supplementary material for: Distinct patterns of brain Fos expression in Carioca High- and Low-conditioned Freezing Rats
Source: PLoS One. 2020 Jul 23;15(7):e0236039. doi: 10.1371/journal.pone.0236039 (PMC7377485; doi:10.1371/journal.pone.0236039)
Supplement: Attachment — Submitted filename: PONE-D_05104.docx [file pone.0236039.s002.docx]

**Summary:** The study attempts to characterize neural circuits that are different between high and low freezing rats. The authors identify a number of substrates that are different between CHL and control and CFL and control rats. What is particularly interesting is the differences that are observed are not necessarily what one would anticipate. This makes the results interesting. However, some concerns need to be addressed before the manuscript is ready for publication.

**Major concern**

1. The strain of rat that is used for CHL and CFL is never mentioned.
2. In the abstract and Introduction the authors need to make it clear that they are examining c-Fos expression in response to contextual fear conditioning. To this end the authors should have a group of rats that were never conditioned or represent baseline differences. How sure are the authors that levels of c-Fos observed do not represent basal differences between the groups of animals?
3. An image of the c-Fos immunohistochemistry needs to be provided
4. The authors do not have enough information to make a model about connectivity between brain regions (e.g. Figures 3 and 4). These figures should be excluded
5. The information presented in the table should be presented in a graph format. This makes it easier to assess the findings
6. Discussion is too long and needs to be tempered. The nature of the results (e.g. no difference in Fos in amygdala brain regions between groups of animals) does not allow for strong statements about the role of these brain regions in trait anxiety.
7. The PL is critical for expression of fear, but most people would not propose that the PL is critical for acquisition of fear memory. Not sure if the citations the authors are providing for this in the Discussion supports this statement.

**Minor concerns**

1. Some references from the Blanchards, Lang, and Davis are missing
2. Better introduction as to why contextual fear conditioning is a good model of anxiety is needed
