# Supplementary material for: Distinct patterns of brain Fos expression in Carioca High- and Low-conditioned Freezing Rats
Source: PLoS One. 2020 Jul 23;15(7):e0236039. doi: 10.1371/journal.pone.0236039 (PMC7377485; doi:10.1371/journal.pone.0236039)
Supplement: Attachment — Submitted filename: RESPONSE TO THE REVIEWER.docx [file pone.0236039.s004.docx]

**RESPONSE TO THE REVIEWER**

Please find a revised manuscript entitled “Distinct patterns of brain Fos expression in Carioca High- and Low-conditioned Freezing Rats" (Manuscript ID: PONE-D-20-05104R2). Once again, we would like to thank the reviewer for calling our attention to the immunostaining photographs.

**RIVEWER #2**

The paper has been greatly improved. The added photomicrographs fill the necessary morphological gap to document the results. The c-fos immunoreaction is fine and nuclei are clearly distinguishable, however I have a concern regarding the background illumination that greatly penalize the immunostaining; some of the photos are gloomy with inhomogenous illumination. Authors should consider to replate the photographs once they have an adequate illumination.

**Response: Done**
